# Microtubules Depolymerization Caused by the CK1 Inhibitor IC261 May Be Not Mediated by CK1 Blockage

**DOI:** 10.1371/journal.pone.0100090

**Published:** 2014-06-17

**Authors:** Martin Stöter, Marc Krüger, George Banting, Doris Henne-Bruns, Uwe Knippschild

**Affiliations:** 1 Department of General and Visceral Surgery, Ulm University Hospital, Ulm, Germany; 2 School of Biochemistry, Medical Sciences Building, University of Bristol, Bristol, United Kingdom; Rush University Medical Center, United States of America

## Abstract

The ubiquitously expressed serine/threonine specific casein kinase 1 (CK1) family plays important roles in the regulation of various physiological processes. Small-molecule inhibitors, such as the CK1δ/ε selectively inhibitor IC261, have been used to antagonize CK1 phosphorylation events in cells in many studies. Here we present data to show that, similarly to the microtubule destabilizing agent nocodazole, IC261 depolymerizes microtubules in interphase cells. IC261 treatment of interphase cells affects the morphology of the TGN and Golgi apparatus as well as the localization of CK1δ, which co-localizes with COPI positive membranes. IC261-induced depolymerization of microtubules is rapid, reversible and can be antagonized by pre-treatment of cells with taxol. At lower concentrations of IC261, mitotic spindle microtubule dynamics are affected; this leads to cell cycle arrest and, depending on the cellular background, to apoptosis in a dose-dependent manner. In addition, FACS analysis revealed that IC261 could induce apoptosis independent of cell cycle arrest. In summary this study provides additional and valuable information about various IC261-induced effects that could be caused by microtubule depolymerization rather than by inhibition of CK1. Data from studies that have used IC261 as an inhibitor of CK1 should be interpreted in light of these observations.

## Introduction

The evolutionary highly conserved, second messenger independent and ubiquitously expressed serine/threonine-specific kinase family CK1 consists in vertebrates of 6 genes (CK1α, δ, ε, γ1-3), which are highly conserved within their kinase domains but differ significantly in their amino acid sequence and length of the N- and C-terminal domains [1], [2].

The steadily increasing number of identified CK1 specific substrates underlines the function of CK1 as an important player in the regulation of many physiological cellular processes, although, so far, not all detected *in* *vitro* substrates have been validated as *in* *vivo* targets. However, a participation of CK1 is known for Wnt signaling [3]–[8], RNA metabolism [9]–[11], circadian rhythm [12]–[14], apoptosis [15]–[19], and DNA repair [20]. Besides these processes members of the CK1 family play a role in chromosome segregation during meiosis [21]–[24], microtubule and spindle dynamics [25]–[28] and membrane transport processes [25], [29]–[32].

Since CK1 plays important roles in many physiological processes a tight regulation of CK1 on different levels is required. At the protein level, autophosphorylation of the CK1δ and CK1ε isoforms results in inhibition of their kinase activities and both cleavage of the C-terminal domain by endoproteases as well as dephosphorylation of autophosphorylation sites leads to elevated kinase activity [33]–[37]. In addition, site specific phosphorylation of CK1δ within its C-terminal domain-mediated by cellular kinases, among them PKA and Chk1 leads to modulation of CK1 activity [38], [39].

Besides posttranslational modifications, subcellular localization and compartmentalization plays an important role in regulating CK1 function. In yeast, CK1 genes Yck1 and Yck2 are anchored by an isoprenyl residue at the inner face of the plasma membrane, whereas Hrr25 primarily localizes within the nucleus via its nuclear localization signal (NLS) [40]. The isoprenylation site and the NLS are essential for biological function and their mutation results in loss of function of the kinase [41], [42]. A chimeric kinase consisting of the kinase domain of Hrr25 and the C-terminal isoprenylation site of Yck2 rescues the Yck1/Yck2 deletion phenotype [41], which stresses the importance of the correct localization for the function of CK1 proteins. In humans CK1δ and CK1ε are localized to the centrosome by the scaffolding protein AKAP450 (A-kinase anchor protein; also termed centrosomal and Golgi N-kinase anchoring protein, CG-NAP) [43]. Furthermore, CK1δ is important for centrosome positioning during T cell activation [28]. In Ewing sarcoma family of tumor (ESFT) cells a chimeric kinase of CK1ε and parts of the C-terminal domain of CK1δ, that is thought to be responsible for centrosome localization, could rescue a CK1δ depletion phenotype [44].

The subcellular localization of CK1 is very important to understand its biological function. At present, reports differ regarding the association of CK1δ with membrane structures, e.g. a co-localization of CK1δ with vesicles segregating from the TGN and with γ-adaptin has been reported [25] as has co-localization with, β’-COP a component of COPI coated vesicles that are responsible for ER-to-Golgi membrane transport [45]. CK1δ has also been shown to be responsible for phosphorylation of ARF GAP1 [32], [46].

Recently it was shown that the CK1δ/ε specific inhibitor IC261 [47] can also act as an inhibitor of microtubule polymerization [48] by directly binding to tubulin, which disrupts spindle formation. Since, in a number of publications IC261 has been used as a CK1δ/ε inhibitor, this publication raises questions about the specificity of IC261 and the interpretation of the reported effects. The situation is complicated by the fact that several studies have suggested that CK1δ/ε could be directly involved in microtubule dynamics. CK1δ co-localizes with spindle microtubules and phosphorylates α- and β-tubulin *in* *vitro* [27], [49]. Furthermore, direct interactions between CK1δ and microtubule associated proteins, such as MAP1A [50], MAP4 [25] and end binding protein 1 (EB1) [28] have been reported.

In the present study, re-investigation of the subcellular localization of CK1δ using high resolution confocal microscopy revealed that CK1δ is located in the perinuclear region close to the TGN and Golgi apparatus, but does not co-localize with these compartments. Instead, CK1δ partly co-localizes with COPI positive membranes and β-COP. Further studies of the IC261-mediated effects on microtubules showed that high concentrations of IC261 disrupt interphase microtubules, finally leading to a dispersed phenotype of perinuclear membranes compartments. This effect of IC261 can be blocked by pretreatment of cells with taxol. Low concentrations of IC261 disrupt spindle microtubules leading to mitotic arrest, post-mitotic arrest or apoptosis. The effect of IC261 on microtubules is reversible. These results are in line with the recent finding that IC261 can act as a microtubule depolymerizing agent. Therefore, the effects on cells induced by IC261 should be interpreted carefully as such effects may be due to either inhibition of CK1 or the depolymerization of microtubules, or a combination of the two.

## Materials and Methods

### Immunofluorescence

Cells used for immunofluorescence microscopy experiments were cultivated on cover slips, washed twice in PBS and fixed in methanol at −20°C. Fixed cells were washed in PBS and blocked using 0.2% gelatin in PBS for 1 h followed by incubation with primary antibodies for 1 h at RT. After washing with PBS, secondary antibodies were incubated for 30 min at RT using fluorophore-labeled IgG. DNA was visualized by DAPI staining (10 min; 0.1 mg/ml). Epifluorescence and time-resolved microscopy was performed on the Olympus IX81 microscope using Cell^R^ imaging and software system (Olympus BioSystems). For time-resolved microscopy cells were cultured in a flow-through chamber (Bioptechs, USA) at 37°C in phenol red-free medium containing with 15 mM HEPES. For confocal microscopy a Leica DM-IRS microscope equipped with a confocal imaging system (TCS NT-Version) was used.

### Flow Cytometry

For flow cytometry analysis, cells cultured in 6-well plates were treated with trypsin (non-adherent cells were previously collected by centrifugation (500 g, 5 min, 4°C)), washed with 4°C PBS +0.1% (w/v) EDTA and fixed in ice-cold 80% ethanol followed by incubation for 15 min on ice and subsequently overnight at −20°C. To perform cell cycle analysis, cells were washed in PBS and stained with 50 µg/ml PI (Sigma) and 10 µg/ml RNase (Fluka) in PBS at 37°C for 30 min. Analysis was performed on a FACScan flow cytometer (Becton Dickinson, Belgium) flow cytometer counting 10000 events per experiment, and for quantification, CellQuest Software was used.

### Cell Culture

For in cell culture experiments the following cell lines were used: Normal rat kidney cells (NRK) [51], immortalized rat fibroblast cells (F111) [52], NRK cells stably expressing GFP-TGN38 (Kplus) [53], *Cercopithecus aethiops* monkey kidneys cells (CV-1) [54] and CV-1 cells stably expressing EYFP-tubulin (see below) which were grown in DMEM. Furthermore we used the human cell line AC1-M88 generated by fusion of extravillous trophoblasts with a choriocarcinoma cell line [55], [56] that was cultured in DMEM/F-12 medium (both Gibco). Media were supplemented with 10% fetal calf serum (FCS; Biochrom) and cells were grown at 37°C in a humidified 5% CO_2_ atmosphere. Where indicated, cells were treated with DMSO, IC261, nocodazole, BFA, taxol or combinations of these and imaged by time-resolved microscopy, fixed for immunofluorescence analysis, or fixed for flow cytometric analysis at the indicated time points. IC261 was synthesized as described by Mashhoon et al. [47].

### Generation of Stable Expressing EYFP-tubulin Cell Line CV-1

Subconfluent CV-1 cells were transfected with Effectene (Qiagen) according to the company’s manual with a vector containing EYFP-tubulin under the control of the CMV promoter (pEYFP-Tub; Clontech). Cells were expanded 1∶5 onto new plates on day 1 and cultivated in medium containing 5 mg/ml geneticin (G418, Invitrogen) from day 2 on after transfection. After 2–3 weeks, geneticin-resistant colonies were analyzed under a fluorescence microscope (Olympus XI81) at 488 nm for expression of EYFP-tubulin. Several colonies (clones) were isolated and expanded, which fulfilled the following criteria: (i) moderate and equal expression level of EYFP-tubulin in all cells, (ii) correct localization of fluorescent protein (microtubule network, mitotic spindle), and (iii) morphology and growth/cell culture behavior similar to the parental cell line CV-1. Clone 5 was chosen for further experiments.

### Michaelis-Menten Kinetics

For further investigation of the interaction of tubulin and CK1 isoforms Michaelis-Menten kinetics were performed. Therefore, a reaction mix was prepared, containing the kinase, as well as the respective substrate, a kinase buffer containing adenosine triphosphate (ATP), and radioactive ^32^P γ-ATP. *In vitro* kinase assays were performed over a time of 30 min at 30°C and separated via SDS-PAGE. Protein bands were visualized by staining with anionic Commassie dye followed by measurement of phosphate incorporation by Cherenkov counting.

### Antibodies

For immuno-detection of CK1δ the monoclonal antibody 128A (IC128A, Icos Corp., USA) or the monoclonal antiserum NC10 [25] was used. The Golgi Apparatus was visualized using the monoclonal antibody 53FC3 against α-mannosidase II (hybridoma supernatant; [57]) or the specific antiserum MG160 against sialogycoprotein MG160, [58] and the TGN was visualized using monoclonal antibody 2F7.1 against TGN38 (hybridoma supernatant; [59]) or antiserum 2268 against TGN38 [60]. COPI vesicles were labeled using a purified polyclonal antibody against coatomer protein β-COP (Ab-1, Oncogene Research, USA).

## Results

**Figure 1:**
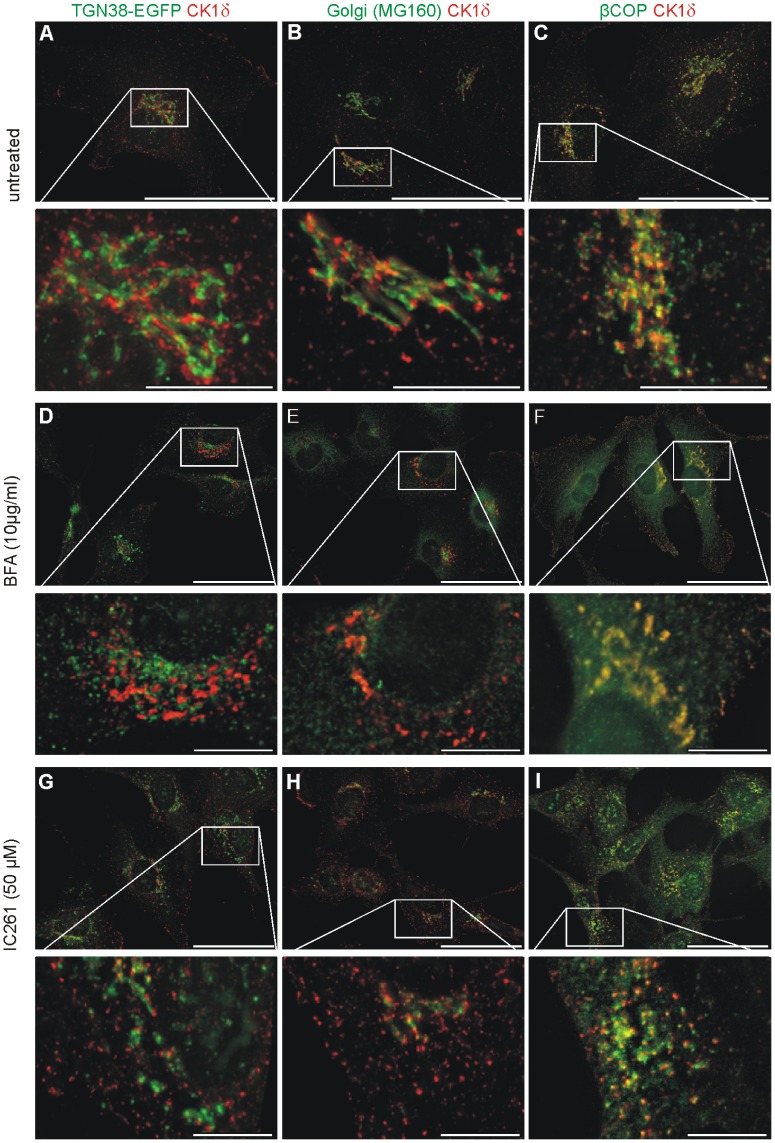
Subcellular association of CK1δ with membrane structures and COPI positive vesicles. NRK cells stably expressing the fusion protein TGN38-EGFP and untransfected NRK cells were either untreated (**A–C**), treated with BFA (10 µg/ml) (**D–F**) or IC261 (50 µM) (**G–I**) and prepared for analysis by immunofluorescence microscopy. The Golgi apparatus was labeled by using a specific antibody (MG160), COPI positive vesicles were labeled with a β-COP specific antibody, and CK1δ was labeled by using the specific antibody 128A.

Several studies, including our own, have described a localization of CK1δ close to intracellular membrane-bound compartments and have deduced a function for CK1δ in membrane transport processes. Since the identity of these membrane-bound compartments is still controversially discussed [25], [45] we re-investigated the subcellular localization of CK1δ with the TGN and the Golgi apparatus (GA) by high resolution fluorescence microscopy. Simultaneous detection of CK1δ and proteins resident in either the TGN or GA in NRK cells, or NRK cells stably expressing EGFP-TGN38, revealed that CK1δ localizes in close proximity to the TGN and GA, but does not co-localize with either of these compartments (figure 1 A, B). These results were confirmed (i) by co-detection of CK1δ using antibodies against an alternative GA-specific marker protein α-mannosidase II and against the TGN-specific marker TGN38, (ii) by using an alternative antibody against CK1δ (NC10, [25]) and (iii) by using another fibroblast cell line (F111) (figure S1). However, we could show a partial co-localization of CK1δ and β-COP/COPI positive membranes (figure 1 C) as suggested previously [45]. To further analyze CK1δ and its localization to perinuclear membrane compartments we treated NRK cells either with Brefeldin A (BFA), the CK1 inhibitor IC261, or the microtubule (MT) destabilizing agent nocodazole and analyzed them by confocal fluorescence microscopy. BFA induced the dissolution of the TGN and the GA resulting in their distribution throughout the cell, while there appeared to be limited change to the distribution of CK1δ positive structures (figure 1 D, E, figure S2 A–L). In agreement with previously published work [61]–[63], BFA causes a redistribution of COPI components to the cytosol (hence the diffuse green signal seen in figure 1 F), but the distribution of CK1δ is apparently unchanged after BFA treatment (figure 1 F).

High concentrations of IC261 (50 µM) did not affect the partial co-localization of CK1δ with β-COP/COPI positive vesicles (figure 1 I). However, IC261 did induce the dissolution of perinuclear TGN and GA structures (figure 1 G, H), with CK1δ retaining a punctate distribution. In fact, the appearance of CK1δ positive structures in IC261 treated cells is somewhat more punctate than in control cells (compare the enlarged images in figure 1 G, H and I with those in A, B and C). Treatment with nocodazole resulted in a similar phenotype (figure S2 M–T) to that of IC261 treated cells.

**Figure 2:**
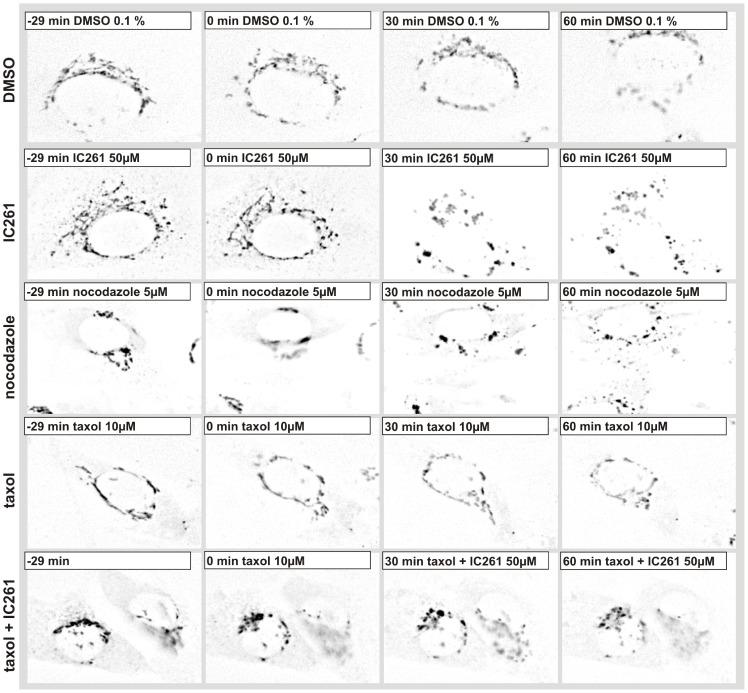
Effect of IC261, nocodazole, taxol, taxol/IC261 on TGN morphology in NRK cells. Line 1–5: NRK cells stably expressing the fusion protein TGN38-EGFP were cultured in a flow-through chamber and observed by time-resolved fluorescence microscopy. At time point “0 min” cell were treated with DMSO (0.1%) **(line 1)**, 50 µM IC261 **(line 2)**, 5 µM nocodazole **(line 3)** or 10 µM taxol **(line 4)**. Here representative cells are shown for the stated time points (see video sequence, movie S1). The solvent DMSO and the treatment with taxol showed no effect on the TGN structure. IC261 as well as nocodazole treatment fragmented the tubular membrane structure of the TGN into vesicles distributed throughout the cell. At time point “−10 min” cells were treated with 10 µM taxol and from time point “0 min” on with 10 µM taxol +50 µM IC261 **(line 5)**. Additional treatment with taxol could prevent the IC261 induced effects on the TGN.

Both, IC261 and nocodazole have been described to bind to the colchicine binding site of α−/β-tubulin heterodimers [48], [64]. Nocodazole stimulates the GTPase activity of tubulin, while IC261 was previously described as an efficient CK1 specific inhibitor [47], [65]. *In vitro,* α- and β-tubulin are phosphorylated by CK1δ at several amino acid residues [25] (and data not shown). Additionally, here we provide evidence from Michaelis-Menten kinetics that CK1δ and CK1ε have a higher affinity towards α-tubulin than to β-tubulin (figure S3). *In vivo* the role of phosphorylation of tubulin in microtubule dynamics is unclear; nevertheless the effects of IC261 and nocodazole on CK1δ localization appear very similar. Therefore, we monitored the effects of IC261 respectively in combination with microtubule stabilizing agent taxol and the effects of IC261, taxol or nocodazole alone on the TGN in NRK cells stably expressing EGFP-TGN38 cells by time-resolved fluorescence microscopy. Cells were imaged for 30 min and DMSO, IC261, taxol or nocodazole were administered at time point “0 min” to the cells via a flow-through chamber and imaged for another 60 min (figure 2, movie S1).

**Figure 3:**
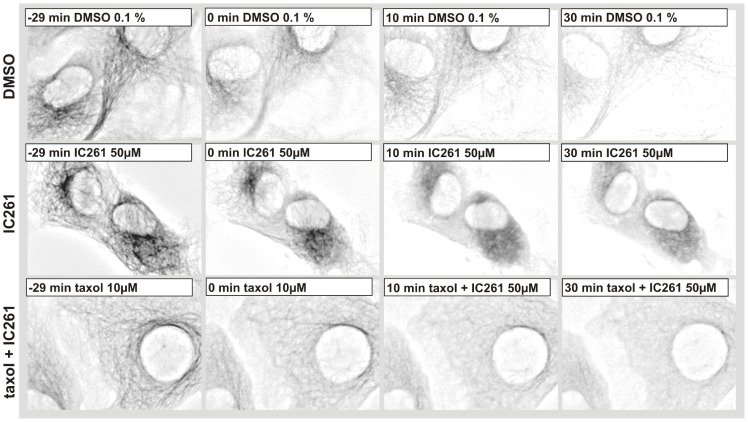
Microtubule destabilizing effect of IC261 in interphase cells. CV-1 cells expressing EYFP-tubulin were cultured in a flow-through chamber, treated with DMSO (0.1%), 50 µM IC261 or 10 µM taxol +50 µM IC261 and observed by time-resolved fluorescence microscopy (see video sequence, movie S2). Here representative cells are shown for time points “−29 min”, “0 min”, “10 min” und “30 min” (**row 1–3**). Treatment with IC261 induced the depolymerization of microtubules within a few minutes (**row 2**) while in cells treated with 10 µM taxol 30 min prior to treatment with IC261 at time point “0 min” the IC261 microtubule destabilizing effect of IC261 could be blocked (**row 3**).

**Figure 4:**
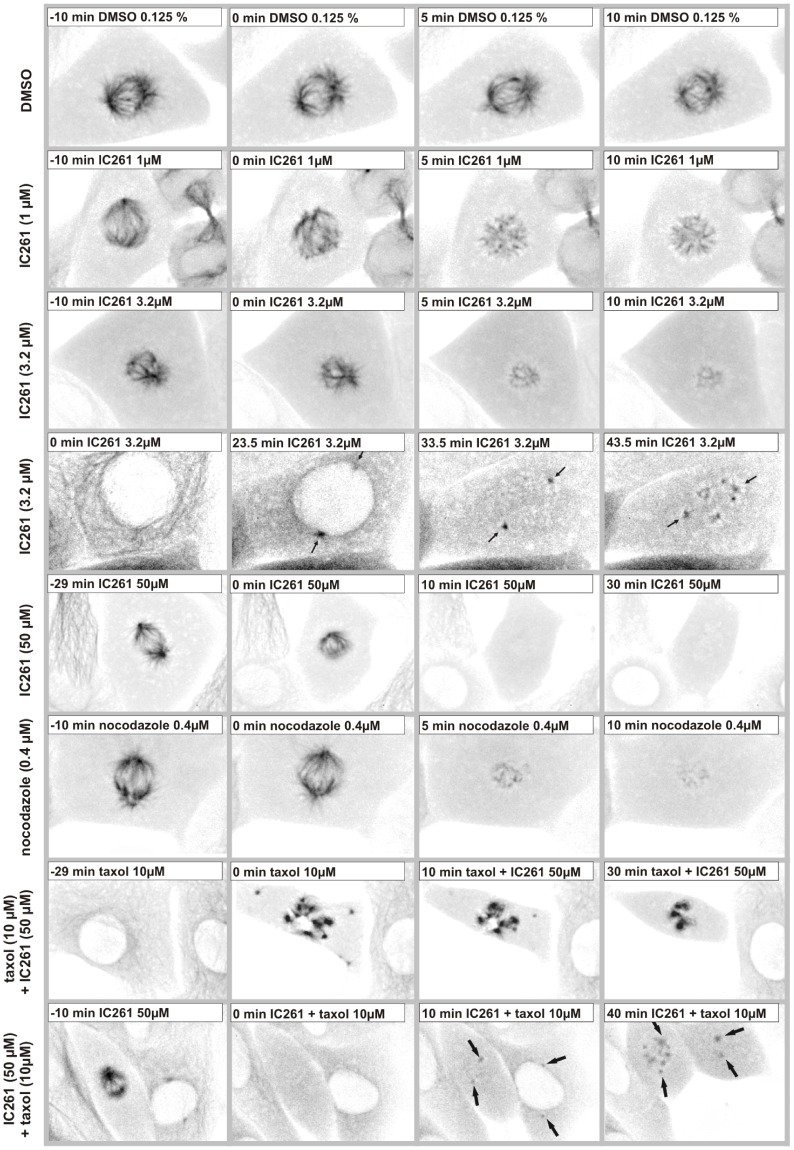
Microtubule destabilizing effect of IC261 in mitotic cells. CV-1 cells expressing EYFP-tubulin were cultured in a flow-through chamber and observed by time-resolved fluorescence microscopy. At time point “0 min” cells were treated with DMSO (0.125%), IC261 (1 µM, 3.2 µM, 50 µM), 10 µM taxol or 0.4 µM nocodazole. Here exemplary cells are shown for indicated time points (see video sequence, movies S3 and S4). Treatment with low concentrations of IC261 induced a depolymerization of spindle microtubules within a few minutes (**row 2–3**) in a concentration dependent manner and interestingly by nocodazole treatment a similar phenotype could be observed (**row 6**). Cells entering mitosis during IC261 treatment had spindle poles and microtubule nucleating centers, but could not form a spindle (**row 4**, arrows indicate spindle poles). Treatment with 50 µM IC261 induced the complete depolymerization of microtubules within a few minutes (3–5 min, **row 5**). When cells were treated with 10 µM taxol during time period “−10 min” to “0 min” prior to treatment with taxol+IC261 at time point “0 min” the MT depolymerizing effect of IC261 could be blocked (**row 7**). When cells were first treated for 10 min with IC261 resulting in a complete dissolution of the spindle apparatus, and subsequently treated with taxol+IC261 tubulin could re-polymerize at the spindle poles (arrows) and in other MT nucleation centers within the cell (**row 8**).

Whereas DMSO (0.1%) and taxol (10 µM) treatment did not show any effects (figure 2 row 1, 4), IC261 (50 µM) and nocodazole induced the fragmentation of TGN38 structures into vesicles within a few minutes (figure 2 row 2–3), most likely due to the previously described microtubule destabilizing effect of IC261 [48]. When cells were treated in parallel with 10 µM taxol the effect of IC261 could be blocked (figure 2 row 5). We then focused on the characterization of IC261 induced alterations of MT structures in interphase and mitotic cells using CV-1 cells stably expressing EYFP-tubulin (CV-1 766CL5); this was done by time-resolved fluorescence microscopy (figure 3, movie S2). In interphase cells the microtubule network was destabilized by treatment of IC261 (50 µM) and dissolved completely within a few minutes (figure 3 row 2). However, combined treatment of cells with IC261 (50 µM) and taxol (10 µM) nearly completely compensated for the MT destabilizing effect of IC261 (figure 3 row 3). To characterize the effects of IC261 on mitotic spindle microtubules at prophase or pro-metaphase, CV-1 766CL5 cells were observed for 30 min before treatment with DMSO, IC261, nocodazole or taxol (time point “0 min”) and then imaged for at least another 60 min (figure 4, movies S3 and S4). Whereas most DMSO (0.125%) treated cells completed mitosis and cytokinesis within approximately 60 min, IC261 treatment affected mitosis through destabilizing mitotic microtubules in a dose-dependent manner. Even at low IC261 concentrations (1 µM) the spindle structure was apparently altered. The spindle apparatus became smaller, the poles were not clearly detectable and spindle dynamics were impaired (figure 4 row 2) finally leading to an arrest of cells in mitosis. At an IC261 concentration of 3.2 µM the spindle apparatus was considerably dissolved after 3–5 min (figure 4 row 3). Cells entering mitosis during IC261 treatment were unable to build up a spindle apparatus (figure 4 row 4), although spindle poles and several MT nucleating centers with short microtubules were recognizable (figure 4 row 4 arrows). While treatment of cells with higher concentrations of IC261 (50 µM) induced the complete dissolution of the spindle apparatus within a few minutes (figure 4 row 5), prior administration of 10 µM taxol (10 min before IC261 addition) blocked the MT destabilizing effect of IC261 even at high doses of 50 µM. Treatment of cells with taxol alone prevented the formation of a functional spindle apparatus and led to an aggregation of polymerized tubulin in the cell center, which could not be antagonized by IC261 (figure 4 row 7). However, if the spindle was completely dissolved by prior treatment of cells with IC261 (50 µM), subsequent treatment with 10 µM taxol could induce the polymerization of tubulin at the centrosomes and later also at several smaller MT nucleation centers (figure 4 row 8). To compare and quantify the dose-dependent MT depolymerizing effects of IC261 and nocodazole, the relative fluorescence intensity of EYFP-tubulin was measured in a defined region of interest (ROI) around the spindle apparatus and outside this region within the cytoplasm. 3.2 µM IC261 resulted in an almost complete dissolution of the mitotic spindle within a few minutes (figure 4 row 3, which could be quantified as a decrease of relative intensity within the ROI spindle and an increase of relative intensity within the ROI cytoplasm due to depolymerized EYFP-tubulin (figure S4 B, D). Similar results were obtained from measurements of cells treated with MT destabilizing agent nocodazole at 0.4 µM (figure 4 row 6; figure S4 C, E). It should be noted that during the observation time no cell treated with IC261 or nocodazole was able to enter anaphase.

**Figure 5:**
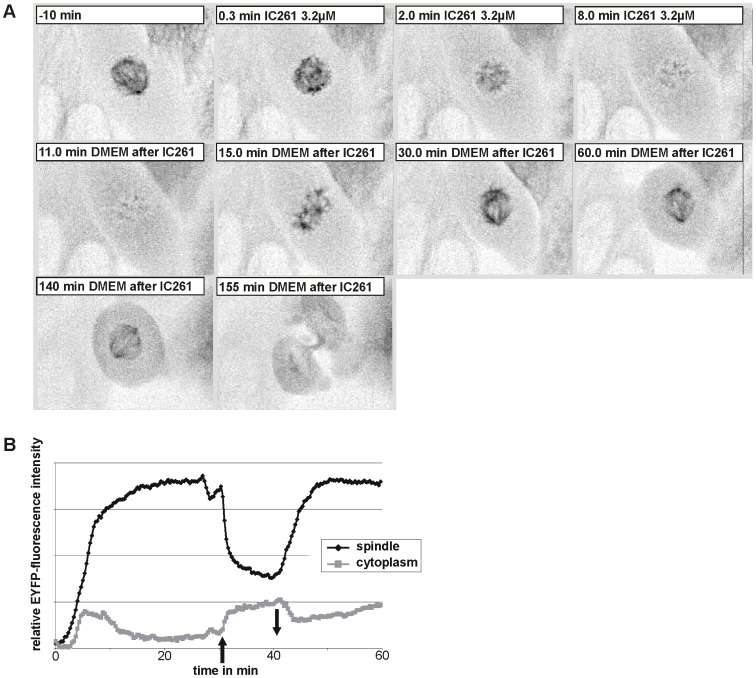
Microtubule depolymerization by IC261 treatment is reversible. (**A**) CV-1 cells expressing EYFP-tubulin were treated at time point “0 min” with 3.2 µM IC261 and observed by time-resolved fluorescence microscopy (see video sequence, movie S5). The spindle apparatus of the representative cell shown here was dissolved within 8 min. At time point “10 min” IC261 was removed by exchange of media. Within a few minutes spindle MTs were built up again (“15 min”) and 20 min after removal a morphologically unimpaired spindle apparatus had been developed (“30 min”). After 2 h the cell proceeded into anaphase and cytokinesis (“155 min”). (**B**) Densitometric analysis of grey values. For quantitative analysis the relative mean intensity of EYFP-tubulin fluorescence signal in a defined region of interest (ROI) around the spindle apparatus and in the cytoplasm was measured by the software CellR. Due to IC261 treatment at time point “0 min” (arrow up) the relative intensity immediately decreased due to MT depolymerization and subsequent removal of IC261 at time point “10 min” (arrow down) lead to a reconstruction of microtubules.

Nocodazole mediated effects are known to be readily reversible [66], [67], raising the question whether this is also the case for IC261. CV-1 766CL5 cells were treated for 10 min with 3.2 µM IC261 and investigated by time-resolved fluorescence microscopy (figure 5, movie S5). Almost immediately after administration of IC261 the spindle apparatus dissolved, but removing IC261 led to the restoration of the spindle apparatus within 20 min (time point “30 min”, figure 5 A). 140 min after removal of IC261 cells completed their cell division by cytokinesis indicating that IC261 induced MT depolymerization and mitotic arrest for a short period of time is reversible.

**Figure 6:**
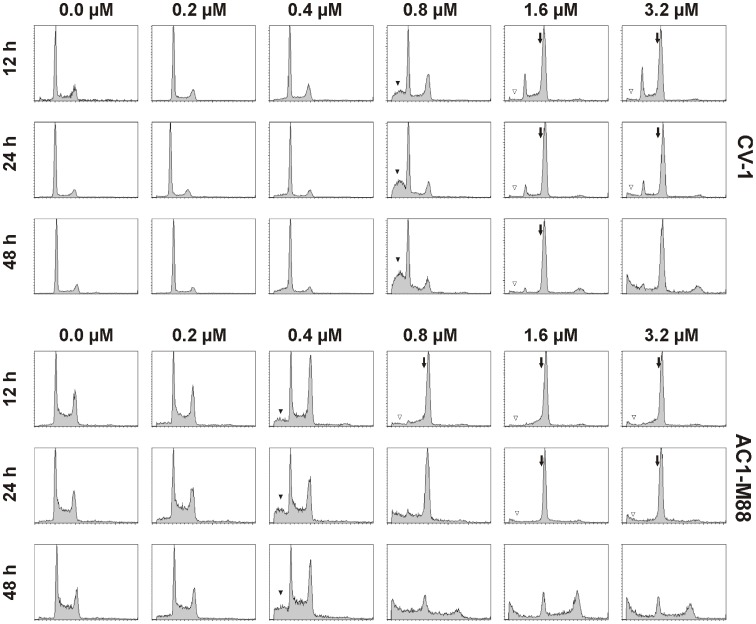
Concentration dependent effects of IC261 on the cell cycle and apoptosis in CV-1 and AC1-M88 cells. CV-1 and AC1-M88 cells were cultivated for 12, 24 and 48 h with different concentrations of IC261 (0.2–3.2 µM) or DMSO (0.0 µM) and subsequently analyzed by FACS analysis with FACScan (Becton Dickinson) (as described in Material and Methods). IC261 induced a full G2/M arrest (arrow) at a cell type dependent concentration (CV1∶1.6 µM, AC1-M88∶0.8 µM). At half of this concentration IC261 induces an increase of the subG1 population (black triangle: CV-1∶0.8 µM, AC1-M88∶0.4 µM). Interestingly, at higher concentrations the amount of cells in subG1 population is smaller indicating less apoptosis (open triangle).

In several cell types inhibition of CK1δ with IC261 has been linked to mitotic arrest and subsequent induction of apoptosis [26], [27], [49]. However CK1 has also been linked to apoptosis directly [16]. Therefore, we re-investigated the cell type specific and dose-dependent effect of IC261 on the cell cycle and on apoptosis at different time points. Here we show, as an example, the concentration dependent effect of IC261 (0.2–3.2 µM) on the cell cycle in the monkey kidney cell line CV-1 and the human trophoblast cell line AC1-M88 for the time points 12 h, 24 h and 48 h using FACS analysis (figure 6). In CV-1 cells it was observed, that the G2/M population was strongly increased after 12 h of treatment with 1.6 µM IC261. An almost complete G2/M arrest was obtained after 48 h for concentrations of 1.6 µM IC261 and higher. In contrast, in AC1-M88 cells 0.8 µM IC261 was sufficient to induce complete G2/M arrest. However between 24 h to 48 h of treatment with IC261 most cells died, probably through apoptosis [27]. Interestingly, in both cell lines an increase of apoptotic cells detectable as a subG1 population was apparent at 12 h at 50% of the concentration needed for completing G2/M arrest (0.8 µM and 0.4 µM, respectively); further referred to as IC_50_(G2/M). Furthermore, it seemed that at higher concentrations than IC_50_(G2/M) the G2/M arrest prevented cells undergoing apoptosis (in CV-1>1.6 µM, in AC1-M88>0.8 µM) at least for the time points 12 h and 24 h. This observation was also seen in several other cell lines (data not shown) and could be a specific apoptotic effect of IC261 at a concentration of IC_50_(G2/M) or a dominant effect of cell cycle arrest suppressing apoptosis. In contrast to these findings treatment of CV-1 and AC1-M88 cells with the CK1 specific inhibitors CK1-7 or D4476 did not alter cell cycle progression and did not induce MT depolymerization (data not shown).

## Discussion

The evolutionary conserved serine/threonine-specific kinase family CK1 is involved in a broad range of intracellular processes and can be regulated by intracellular compartmentalization. We here provide evidence that CK1δ is localized at perinuclear membrane compartments and co-localizes with β-COP, a subunit of the coatomer protein complex coating COPI vesicles. Treatment of cells with the CK1-inhibitor IC261 induces changes in CK1δ localization as well as changes of other membrane compartments such as the TGN and Golgi apparatus, most likely due to depolymerization of microtubules. The aim of the present study was to unravel the various effects of IC261 described in recent years on CK1δ, on microtubule dynamics, and on membrane transport processes.

Since it has been reported that CK1δ is localized on several intracellular membrane compartments, e.g. TGN [25] or GA [45], we investigated the subcellular localization of CK1δ by fluorescence microscopy at high resolution and found that CK1δ neither co-localizes with the TGN nor GA structures, but is in close proximity to both compartments. This finding was confirmed by using multiple antibodies for CK1δ and for typical TGN and GA markers in two rat cell lines. Whereas the GA and TGN compartments looked like the well-known stack of cisternae, CK1δ-positive structures appeared more vesicular and in close proximity to the TGN and GA. Furthermore CK1δ seemed to be closer to the GA markers than the TGN marker (figure 2 A–D and Q–T). Interestingly, CK1δ showed partial co-localization with β-COP positive vesicles. β-COP is a subunit of the coatomer complex coating COPI vesicles, which are responsible for retrograde GA-to-ER or intra-GA membrane transport processes [68], [69]. The hypothesis that CK1δ could be involved in GA-ER transport is supported by (i) CK1δ co-localizes with another coatomer protein β’-COP [45], and (ii) by the report of CK1δ regulating membrane binding of ARF GAP1 [32] - a protein stimulating GTPase activity of ARF1, which is required for the uncoating of COPI vesicles. However, in the latter report IC261 was used at high concentration (50–200 µM) for experiments in cells. The authors argue that *in* *vitro* experiments use a lower ATP concentration (10 µM), whereas intracellular ATP concentrations *in* *vivo* are higher (∼3 mM). Thus the described IC_50_ value for *in* *vitro* experiments (1.0±0.3 µM) [47] has to be adapted for cellular assays. Recently it was shown that IC261 depolymerizes spindle MTs at low concentrations (1 µM) and inhibits *in* *vitro* microtubule polymerization (at 3–10 µM) by binding directly to the colchicine binding site [48]. This is in line with our findings that IC261 affects spindle dynamics in the low micro molar range (1–3.2 µM) and depolymerizes interphase MTs at higher concentrations (∼50 µM). Interestingly, a recent study [70] on structure-activity relationship towards a proposed inhibitory effect of IC261 against γ-secretase [71] showed that cellular activity of IC261 and its activity in cell-free assays is inconsistent. Three analogues of IC261 lost their ability to inhibit CK1 kinase activity *in* *vitro*, but maintained an inhibitory activity on γ-secretase in cellular assays; these findings suggest an off-CK1-target activity of IC261 and could be related to IC261 induced MT depolymerization. This is supported by the observation that treatment with other CK1 specific inhibitors, CK1-7 and D4476 (our own unpublished data) or PF670462 [48], did not alter MT morphology or cell cycle progression.

Nevertheless, Yu and Roth show convincing results to describe a role of CK1δ in membrane binding of ARF GAP1 using other tools such as an inhibitory antibody against CK1δ and expression of dominant negative CK1δ [32]. However, conclusions drawn from cellular assays using high concentrations of IC261 (10–200 µM) should be carefully reviewed. MT depolymerization through IC261 produces a similar phenotype to that generated by nocodazole, as seen for the GA and TGN (figure 2), as well as for spindle and interphase MTs (figure 4, and data not shown, respectively). Still, the phenotype of fragmentation of perinuclear membrane-bound compartments by IC261 can be prevented by pre-treatment with taxol. However, interphase microtubules could be partially stabilized through pre- and co-treatment with taxol (figure 3), nevertheless our study also shows that in cells treated with taxol and IC261 MT dynamics are severely disturbed, and one cannot assume unaffected MT dynamics as suggested in a recent report [30]. In highly dynamic processes like the formation of mitotic spindles, neither pre- nor post-treatment with taxol could rescue the effect of IC261 on MTs (figure 5). Finally, the MT depolymerization effects of IC261 are reversible, similarly as shown for nocodazole [67], [72]).

Although the results presented here, and those of others [48], strongly suggest that IC261 has an effect as a MT depolymerizing agent, we cannot rule out the possibility that CK1 proteins could participate in the regulation of MT dynamics. Several studies have reported that members of the CK1 family, especially CK1δ associate and/or phosphorylate tubulin and MT associated proteins (MAPs) [25], [28], [50], [73]–[75] and are thereby likely to be involved in the regulation of MT dynamics. Several studies have also shown that CK1δ, independent of IC261, is localized at the centrosome [25], [27], [45] and that CK1δ controls centrosome positioning and neurite outgrowth [28], [44]. A recent report describes a role for CK1δ at the centrosome in ciliogenesis, MT nucleation and Golgi organization; this appears to be through a direct interaction with AKAP450 [76].

Former studies have shown a direct linkage between CK1 and apoptosis following mitotic arrest by treatment with the CK1 inhibitor IC261 [26], [27], [49]. IC261 was used in low concentrations (0.6–1.2 µM), no effects on interphase MTs were detected in cells and phenotypes on the mitotic spindle were dissimilar to those resulting from nocodazole treatment. However, these results could also result from the MT depolymerizing effect of IC261, dependent on the chosen cell line or on time and concentration of the chosen IC261 treatment. Therefore, we re-investigated the dose-dependent effects of IC261 on the cell cycle and on apoptosis at different time points and in several cell types. It is already known that IC261 mediated effects are dependent on the p53 status. Mouse embryo fibroblasts containing active p53 arrest in the post-mitotic G1 phase (4N) when treated with IC261, whilst cells expressing a non-functional p53 undergo post-mitotic endoreduplication (8N) [49]. These findings were confirmed in extravillous trophoblast hybrid cells where the cellular p53 background was found to play an important role in the induction of apoptosis upon IC261 treatment [27]. Additionally, recent reports revealed that upregulated myc expression sensitizes cells for therapeutics targeting CK1ε [77]. In our studies, IC261 induced a transient full G2/M arrest at a cell type dependent concentration. We have also shown that at half of this concentration (IC_50_(G2/M)) the subG1 population increases (figure 6). This increase of apoptotic cells cannot be due to G2/M arrest, because at IC_50_(G2/M) the population of 4N cells is not significantly increased. It was shown, that CK1 phosphorylates Bid and thereby prevents cleavage by caspase 8 [16]. Therefore an inhibition of CK1 at low concentrations of IC261 could lead to activation of pro-apoptotic protein Bid and thereby to increased apoptosis indicated by increased subG1 population. However, it remains unclear why at higher concentrations of IC261 the cell cycle arrest at G2/M is dominant over the pro-apoptotic effect.

In summary this study provides data that extends the knowledge of IC261 induced effects in cells. We demonstrate that the CK1 kinase inhibitor IC261 mediates off-CK1-target effects by depolymerizing MTs in a dose-dependent and reversible manner. Therefore, results of previous studies using IC261 as a CK1 inhibitor should be interpreted carefully. Here, we also present evidence that CK1 is neither localized at the TGN nor at the GA, but co-localizes with the COPI protein β-COP.

## Supporting Information

Figure S1 **Perinuclear localization of CK1δ in NRK and F111 cells.** NRK and F111 were prepared for immunofluorescence microscopy as described in Material and Methods and incubated with antibodies 2268 or 2F7.1 specific for the TGN marker protein TGN38 (green) **(A)** or the antibodies 53FC3 or MG160 specific for the Golgi marker protein α-mannosidase (green) **(B)**. In addition, CK1δ was stained using specific antibodies 128A or NC-10 (red). Examination of the merged image clearly demonstrates that CK1δ localizes in close proximity to the Golgi apparatus and the TGN.
(TIF)

Figure S2 **Perinuclear localization of CK1δ and effect of BFA and nocodazole.** NRK cells stably expressing the fusion protein TGN38-EGFP were either untreated or treated with 5 µg/ml BFA or 0.4 µM nocodazole for indicated time points and the Golgi apparatus and CK1δ were immunofluorescently labeled using specific antibodies MG160 and 128A, respectively. BFA treatment induced the dissolution of membrane structures and treatment with nocodazole resulted in a fragmentation of perinuclear membrane structures, however TGN-, Golgi- and CK1δ-markers stayed in close proximity. A similar phenotype was observed in IC261 treated cells.
(TIF)

Figure S3 **CK1 mediated phosphorylation of tubulin** ***in*** ***vitro*** **.** Recombinant human α- or β-tubulin was phosphorylated by the indicated CK1 isoform and analyzed by Michaelis-Menten kinetics. Increasing concentrations of GST-tubulin were phosphorylated for 30 min at 30°C with the stated CK1 isoform in the presence of radioactive ^32^P-γATP and incorporated ^32^P measured by Cherenkov counting. Error bars indicate standard error of the mean, CK1δ (CK1δ from rat as GST-tagged fusion protein), CK1δ KD (kinase domain of CK1δ), CK1ε (CK1ε from human as HIS-tagged fusion protein), n indicates the number of independent performed experiments. The comparison of K_m_ values revealed a higher affinity of CK1 isoforms for α-tubulin than β-tubulin. K_m_-values: **α-tubulin1B** 204.9+/−72.2 nM (CK1δ), 266.2+/−64.1 nM (CK1δ KD), 329.2+/−203.0 nM (CK1ε); **α-tubulin4A** 336.7+/−32.0 nM (CK1δ), 438.3+/−20.8 nM (CK1δ KD), 602.4+/−36.7 nM (CK1ε); **β-tubulin2A** 1167.5+/−106.6 nM (CK1δ), 1465.3+/−139.7 nM (CK1δ KD), 1408.7+/−354.9 nM (CK1ε). Significance was calculated by performing an unpaired t-test with GraphPad Prism 6 (* = p<0.05, ** = p<0.01, *** = p<0.001).
(TIF)

Figure S4 **Quantitative analysis of microtubule destabilizing effect of IC261.** CV-1 cells expressing EYFP-tubulin were cultured in a flow-through chamber, observed by time-resolved fluorescence microscopy every 30 sec and were treated with DMSO (0.125%) **(A)**, 1 µM **(B)** and 3.2 µM IC261**(C)** or 0.1 µM **(D)** and 0.4 µM nocodazole **(E)** at indicated time points (arrow). For quantitative analysis of polymerized tubulin in the spindle the relative grey level of EYFP-tubulin fluorescence signal was measured by the software CellR. In brief, for a defined region of interest (ROI) around the spindle apparatus (ROI-SA), in an adjacent area in the cytoplasm (ROI-C) and in an image region with no cells (ROI-bkg) the mean intensity was measured and ROI-SA minus ROI-bkg and ROI-C minus ROI-bkg was computed over time as relative grey value. In the beginning of mitosis during formation of the spindle the fluorescence signal increases in ROI-SA, while the signal in ROI-C decreases. IC261 as well as nocodazole lead to a depolymerization of MTs and decrease of the fluorescence intensity within a few minutes (3–5 min) in a concentration dependent manner. At the same time the relative grey levels of ROI-C increased due to the depolymerized EYFP-tubulin.
(TIF)

Movie S1 **Effect of IC261, nocodazole, taxol or taxol/IC261 in TGN morphology in NRK cells.** NRK cells stably expressing the fusion protein TGN38-EGFP were cultured in a flow-through chamber and observed by time-resolved fluorescence microscopy. At time point “0 min” cell were treated with DMSO (0.1%), 50 µM IC261, 5 µM nocodazole or 10 µM taxol. Here exemplary cells are shown for the stated time points. The solvent DMSO and the treatment with taxol showed no effect on the TGN structure. IC261 as well as nocodazole treatment fragmented the tubular membrane structure of the TGN into vesicles distributed throughout the cell. Line 6–7: At time point “−10 min” K* cells were treated with 10 µM taxol and from time point “0 min” on with 10 µM taxol +50 µM IC261. Additional treatment with taxol could prevent the IC261 induced effects on the TGN.
(AVI)

Movie S2 **Microtubule destabilizing effect of IC261 in interphase cells.** CV-1 cells expressing EYFP-tubulin were cultured in a flow-through chamber, treated with DMSO (0.1%), 50 µM IC261 or 10 µM taxol +50 µM IC261 and observed by time-resolved fluorescence microscopy. Here exemplary cells are shown for time points “−29 min”, “0 min”, “10 min” und “30 min”. Treatment with IC261 induced the depolymerization of microtubules within a few minutes while in cells treated with 10 µM taxol 30 min prior to treatment with IC261 at time point “0 min” the IC261 microtubule destabilizing effect of IC261 could be blocked.
(AVI)

Movie S3 **Microtubule destabilizing effect of IC261 and nocodazole in mitotic cells.** CV-1 cells expressing EYFP-tubulin were cultured in a flow-through chamber and observed by time-resolved fluorescence microscopy. At time point “0 min” cells were treated with DMSO (0.125%), IC261 (1 µM, 3.2 µM) or 0.4 µM nocodazole. Here exemplary cells are shown for indicated time points. Treatment with low concentrations of IC261 induced a depolymerization of spindle microtubules within a few minutes in a concentration dependent manner and interestingly by nocodazole treatment a similar phenotype could be observed. Cells entering mitosis during IC261 treatment had spindle poles and microtubule nucleating centers, but could not form a spindle.
(AVI)

Movie S4 **Microtubule destabilizing effect of IC261 in mitotic cells.** CV-1 cells expressing EYFP-tubulin were cultured in a flow-through chamber and observed by time-resolved fluorescence microscopy. At time point “0 min” cells were treated with DMSO (0.125%), 50 µM IC261 and/or 10 µM taxol. Here exemplary cells are shown for indicated time points. Treatment with 50 µM IC261 induced the complete depolymerization of microtubules within a few minutes (3–5 min). When cells were treated with 10 µM taxol during time period “−10 min” to “0 min” prior to treatment with taxol+IC261 at time point “0 min” the MT depolymerizing effect of IC261 could be blocked. When cells were first treated for 10 min with IC261 resulting in a complete dissolution of the spindle apparatus, and subsequently treated with taxol+IC261 tubulin could re-polymerize at the spindle poles and in other MT nucleation centers within the cell.
(AVI)

Movie S5 **Microtubule depolymerization by IC261 treatment is reversible.** CV-1 cells expressing EYFP-tubulin were treated at time point “0 min” with 3.2 µM IC261 and observed by time-resolved fluorescence microscopy. The spindle apparatus of the exemplary cell shown here was dissolved within 8 min. At time point “10 min” IC261 was removed by exchange of media. Within a few minutes spindle MTs were built up again (“15 min”) and 20 min after removal a morphologically unimpaired spindle apparatus had been developed (“30 min”). After 2 h the cell proceeded into anaphase and cytokinesis (“155 min”).
(AVI)
