# Supplementary figures and images for: Microtubules Depolymerization Caused by the CK1 Inhibitor IC261 May Be Not Mediated by CK1 Blockage
Source: PLoS One. 2014 Jun 17;9(6):e100090. doi: 10.1371/journal.pone.0100090 (PMC4061085; doi:10.1371/journal.pone.0100090)

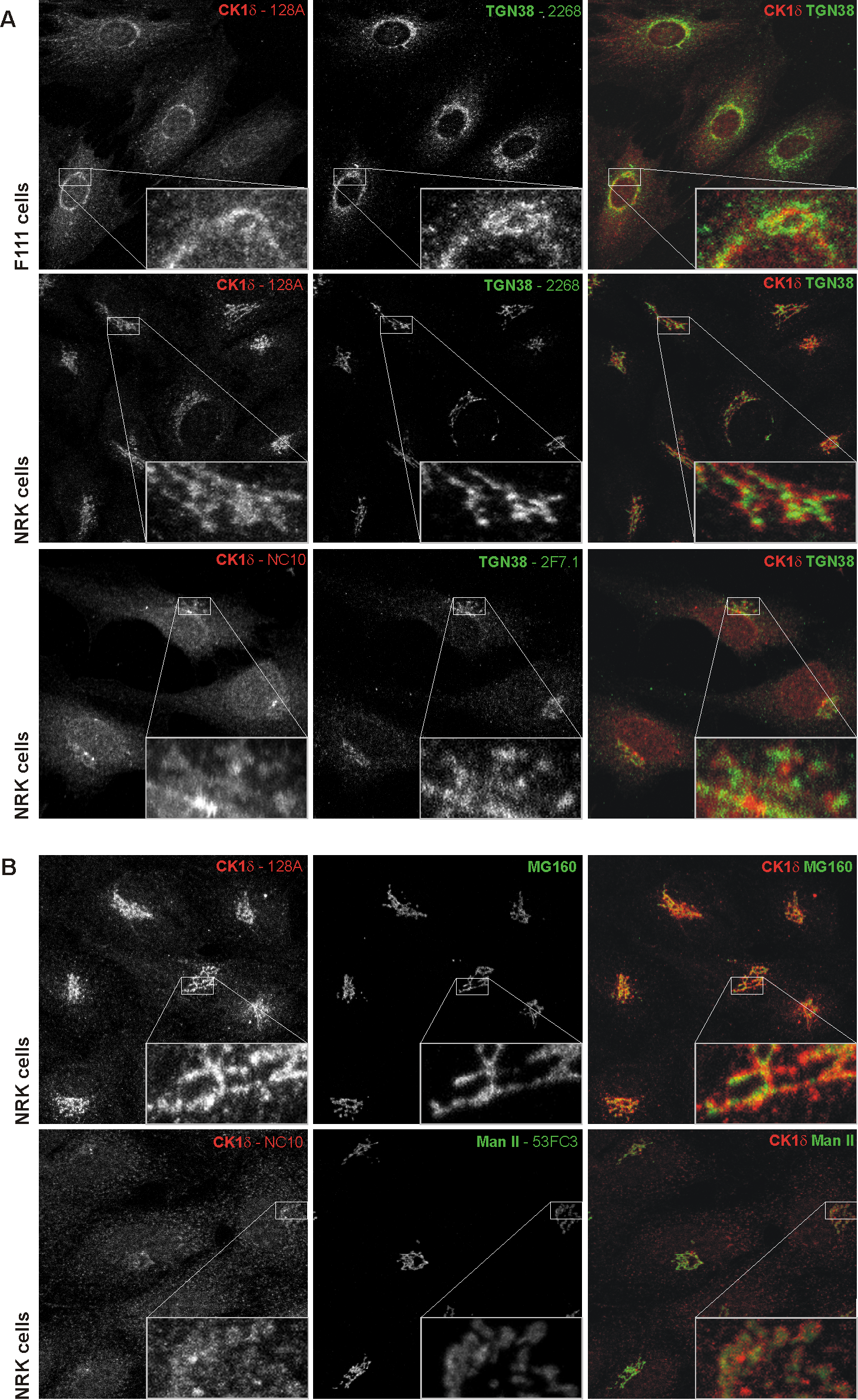

Supplement: Figure S1 — Perinuclear localization of CK1δ in NRK and F111 cells. NRK and F111 were prepared for immunofluorescence microscopy as described in Material and Methods and incubated with antibodies 2268 or 2F7.1 specific for the TGN marker protein TGN38 (green) (A) or the antibodies 53FC3 or MG160 specific for the Golgi marker protein α-mannosidase (green) (B). In addition, CK1δ was stained using specific antibodies 128A or NC-10 (red). Examination of the merged image clearly demonstrates that CK1δ localizes in close proximity to the Golgi apparatus and the TGN. (TIF) [file pone.0100090.s001.tif]

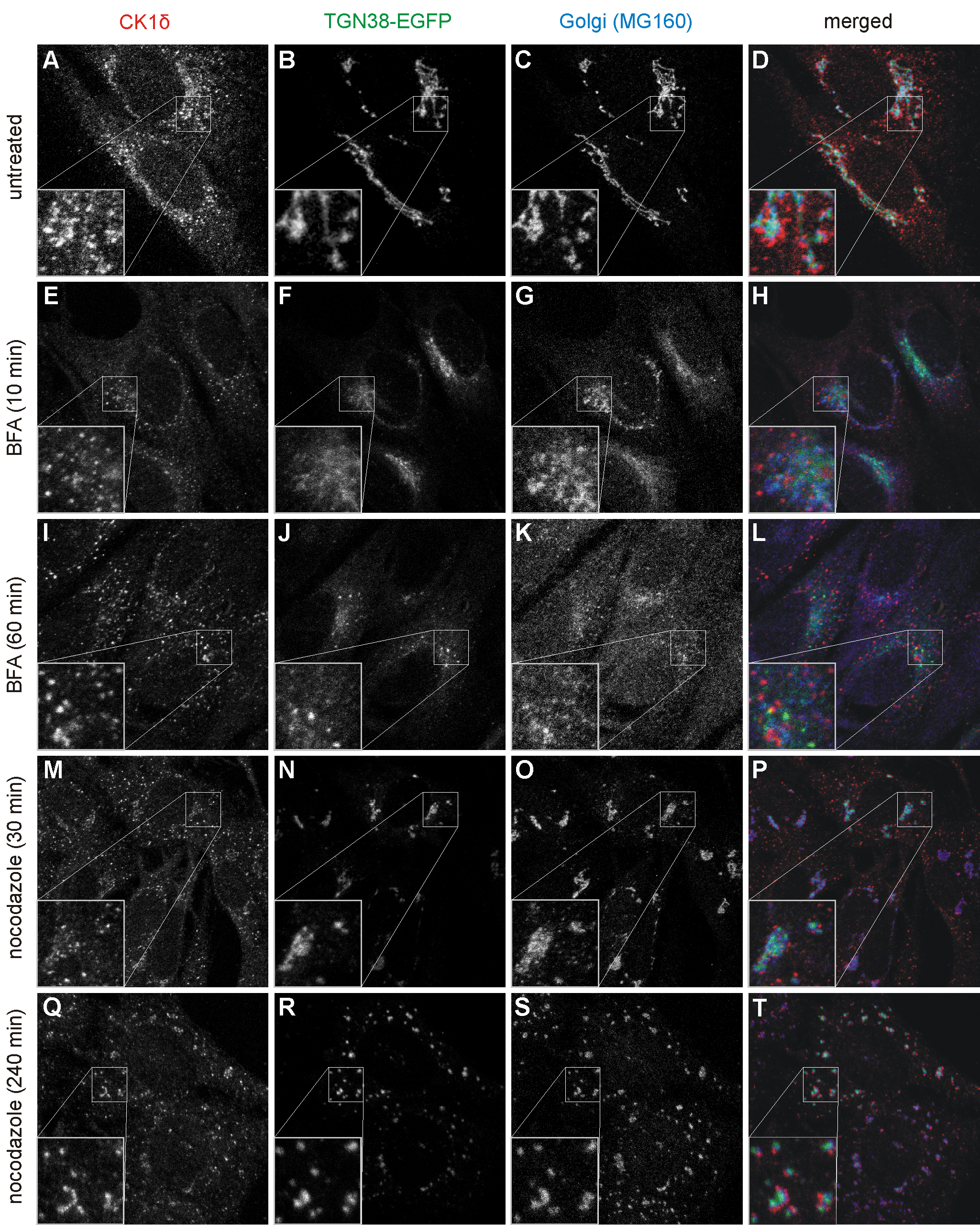

Supplement: Figure S2 — Perinuclear localization of CK1δ and effect of BFA and nocodazole. NRK cells stably expressing the fusion protein TGN38-EGFP were either untreated or treated with 5 µg/ml BFA or 0.4 µM nocodazole for indicated time points and the Golgi apparatus and CK1δ were immunofluorescently labeled using specific antibodies MG160 and 128A, respectively. BFA treatment induced the dissolution of membrane structures and treatment with nocodazole resulted in a fragmentation of perinuclear membrane structures, however TGN-, Golgi- and CK1δ-markers stayed in close proximity. A similar phenotype was observed in IC261 treated cells. (TIF) [file pone.0100090.s002.tif]

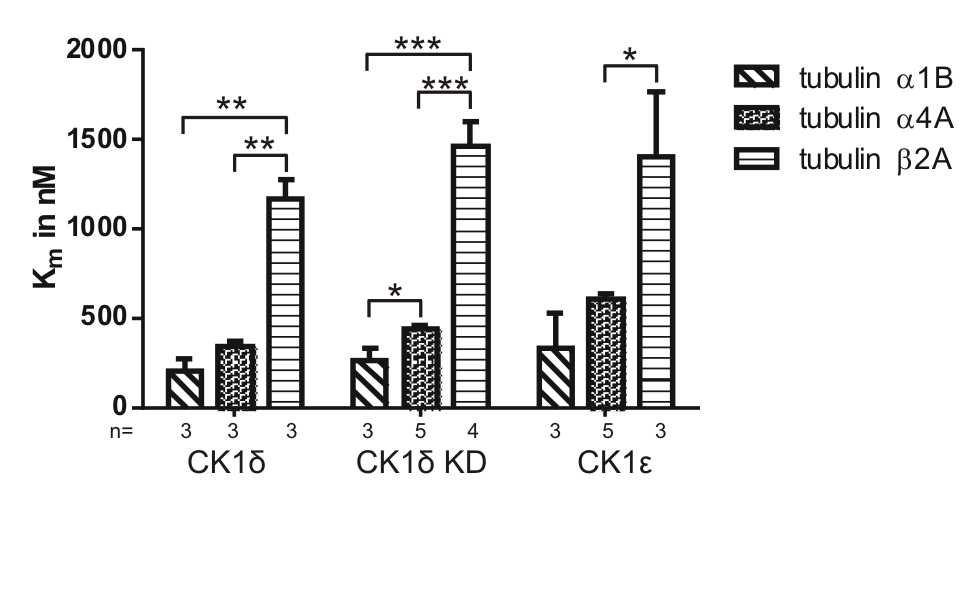

Supplement: Figure S3 — CK1 mediated phosphorylation of tubulin in vitro . Recombinant human α- or β-tubulin was phosphorylated by the indicated CK1 isoform and analyzed by Michaelis-Menten kinetics. Increasing concentrations of GST-tubulin were phosphorylated for 30 min at 30°C with the stated CK1 isoform in the presence of radioactive 32P-γATP and incorporated 32P measured by Cherenkov counting. Error bars indicate standard error of the mean, CK1δ (CK1δ from rat as GST-tagged fusion protein), CK1δ KD (kinase domain of CK1δ), CK1ε (CK1ε from human as HIS-tagged fusion protein), n indicates the number of independent performed experiments. The comparison of Km values revealed a higher affinity of CK1 isoforms for α-tubulin than β-tubulin. Km-values: α-tubulin1B 204.9+/−72.2 nM (CK1δ), 266.2+/−64.1 nM (CK1δ KD), 329.2+/−203.0 nM (CK1ε); α-tubulin4A 336.7+/−32.0 nM (CK1δ), 438.3+/−20.8 nM (CK1δ KD), 602.4+/−36.7 nM (CK1ε); β-tubulin2A 1167.5+/−106.6 nM (CK1δ), 1465.3+/−139.7 nM (CK1δ KD), 1408.7+/−354.9 nM (CK1ε). Significance was calculated by performing an unpaired t-test with GraphPad Prism 6 (* = p<0.05, ** = p<0.01, *** = p<0.001). (TIF) [file pone.0100090.s003.tif]

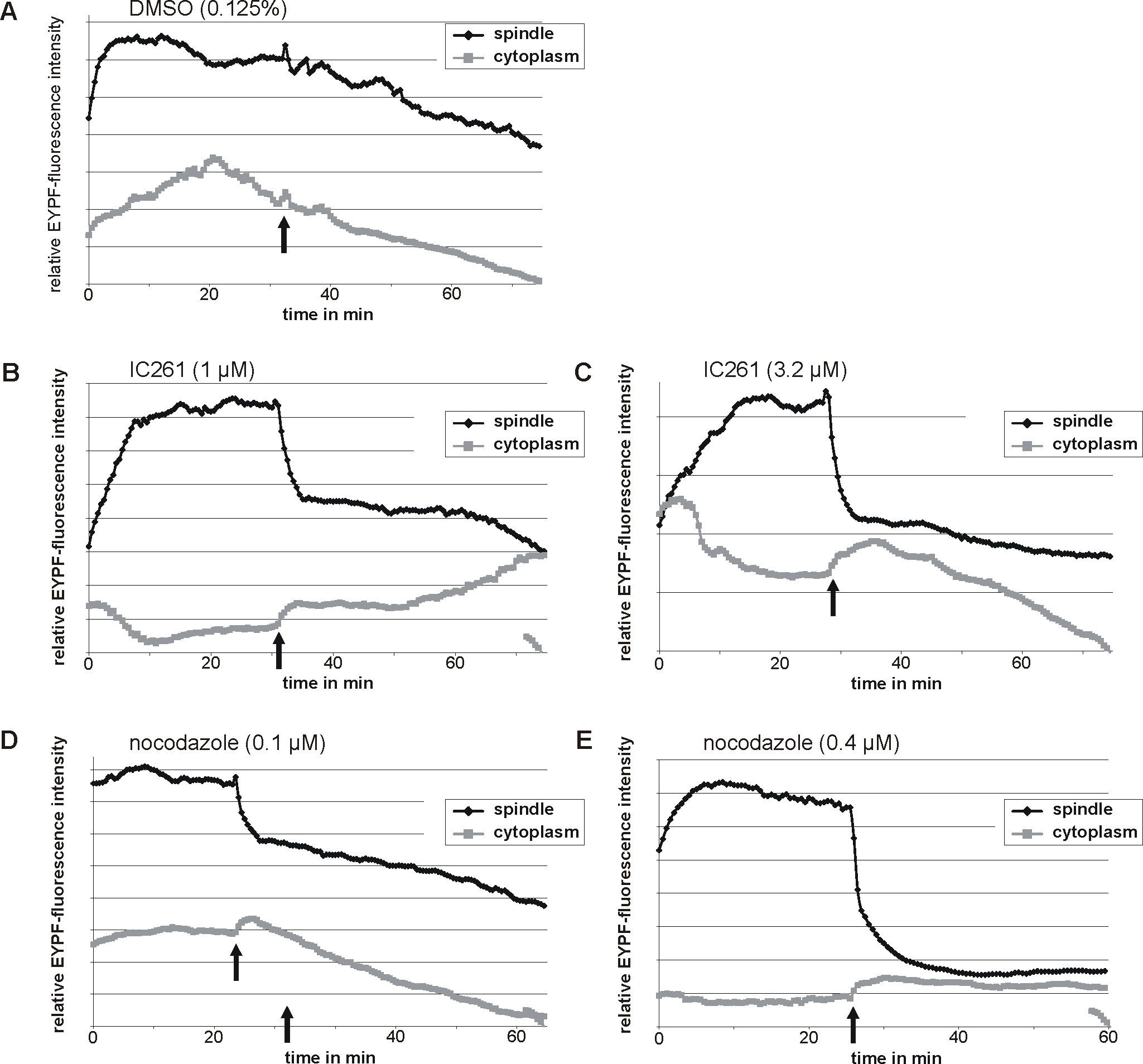

Supplement: Figure S4 — Quantitative analysis of microtubule destabilizing effect of IC261. CV-1 cells expressing EYFP-tubulin were cultured in a flow-through chamber, observed by time-resolved fluorescence microscopy every 30 sec and were treated with DMSO (0.125%) (A), 1 µM (B) and 3.2 µM IC261(C) or 0.1 µM (D) and 0.4 µM nocodazole (E) at indicated time points (arrow). For quantitative analysis of polymerized tubulin in the spindle the relative grey level of EYFP-tubulin fluorescence signal was measured by the software CellR. In brief, for a defined region of interest (ROI) around the spindle apparatus (ROI-SA), in an adjacent area in the cytoplasm (ROI-C) and in an image region with no cells (ROI-bkg) the mean intensity was measured and ROI-SA minus ROI-bkg and ROI-C minus ROI-bkg was computed over time as relative grey value. In the beginning of mitosis during formation of the spindle the fluorescence signal increases in ROI-SA, while the signal in ROI-C decreases. IC261 as well as nocodazole lead to a depolymerization of MTs and decrease of the fluorescence intensity within a few minutes (3–5 min) in a concentration dependent manner. At the same time the relative grey levels of ROI-C increased due to the depolymerized EYFP-tubulin. (TIF) [file pone.0100090.s004.tif]
